# Advances in Research on the Effects and Mechanisms of Chemokines and Their Receptors in Cancer

**DOI:** 10.3389/fphar.2022.920779

**Published:** 2022-06-13

**Authors:** Jing Xu, Jing-quan Li, Qi-lei Chen, Elena A. Shestakova, Vsevolod A. Misyurin, Vadim S. Pokrovsky, Elena M. Tchevkina, Hu-biao Chen, Hang Song, Jian-ye Zhang

**Affiliations:** ^1^ Guangzhou Municipal and Guangdong Provincial Key Laboratory of Molecular Target & Clinical Pharmacology, NMPA and State Key Laboratory of Respiratory Disease, School of Pharmaceutical Sciences and the Fifth Affiliated Hospital, Guangzhou Medical University, Guangzhou, China; ^2^ Department of Biochemistry and Molecular Biology, School of Integrated Chinese and Western Medicine, Anhui University of Chinese Medicine, Hefei, China; ^3^ The First Affiliated Hospital, Hainan Medical University, Haikou, China; ^4^ School of Chinese Medicine, Hong Kong Baptist University, Hong Kong, China; ^5^ N.N. Blokhin National Medical Research Center of Oncology of the Ministry of Health of the Russian Federation, Moscow, Russia; ^6^ Department of Biochemistry, People’s Friendship University, Moscow, Russia

**Keywords:** chemokine, chemokine receptor, epigenetic regulation, molecular mechanisms, combination medication

## Abstract

Cancer is a common and intractable disease that seriously affects quality of life of patients and imposes heavy economic burden on families and the entire society. Current medications and intervention strategies for cancer have respective shortcomings. In recent years, it has been increasingly spotlighted that chemokines and their receptors play vital roles in the pathophysiology of cancer. Chemokines are a class of structurally similar short-chain secreted proteins that initiate intracellular signaling pathways through the activation of corresponding G protein-coupled receptors and participate in physiological and pathological processes such as cell migration and proliferation. Studies have shown that chemokines and their receptors have close relationships with cancer epigenetic regulation, growth, progression, invasion, metastasis, and angiogenesis. Chemokines and their receptors may also serve as potential targets for cancer treatment. We herein summarize recent research progresses on anti-tumor effects and mechanisms of chemokines and their receptors, suggesting avenues for future studies. Perspectives for upcoming explorations, such as development of multi-targeted chemokine-based anti-tumor drugs, are also discussed in the present review.

## Introduction

According to the latest statistics from American Cancer Society, the number of cancer cases and deaths remains high throughout the years, and has been even elevated due to delayed diagnosis under the COVID-19 pandemic (Siegel et al., 2022). The whirlwind growth of economy and technology have driven major development of the research on cancer, especially on the pathogenic mechanisms and relevant therapeutic strategies. Among the research topic on cancer, the study of tumor microenvironment stands out due to its close relation to the occurrence, growth, and metastasis of cancer. Tumor microenvironment not only affect the tumor cells themselves, but also the surrounding cells (fibroblasts, immune and inflammatory cells, and glial cells), as well as the interstitial cells, microvasculature, and biomolecules that penetrating into nearby tissues (Hinshaw and Shevde 2019; Anderson and Simon 2020; DeBerardinis 2020). Recent studies have shown that the chemokine family is considered closely related to tumor microenvironment. Chemokines are a subfamily of small-molecule cytokines secreted by cells and play an essential role in transportation of immune cells and development of lymphoid tissue, with the function of inducing targeted chemotaxis of neighboring responding cells (van der Vorst et al., 2015; Hughes and Nibbs 2018). To date, 48 different chemokines have been reported, and they can be classified into four classes (C, CC, CXC and CX3C) depending on the number and location of their amino-terminal (N-terminal) pre-cysteines. They exert the biological function through selective binding to their receptors, which are G protein-coupled receptors (GPCRs) expressed on various chemotactic immune cells in tissues, organs, and circulatory system (Miller and Mayo 2017). Recent studies have shown that chemokines and their receptors also serve an important purpose in the induction of immune cells against tumors (Chow and Luster 2014; Nagarsheth et al., 2017). Based on this, the present review focuses on the study of the anti-tumor activity and mechanisms of anti-cancer effect of chemokines, with the aim of providing reference for future research.

## Structure and Function of Chemokines

**FIGURE 1:**
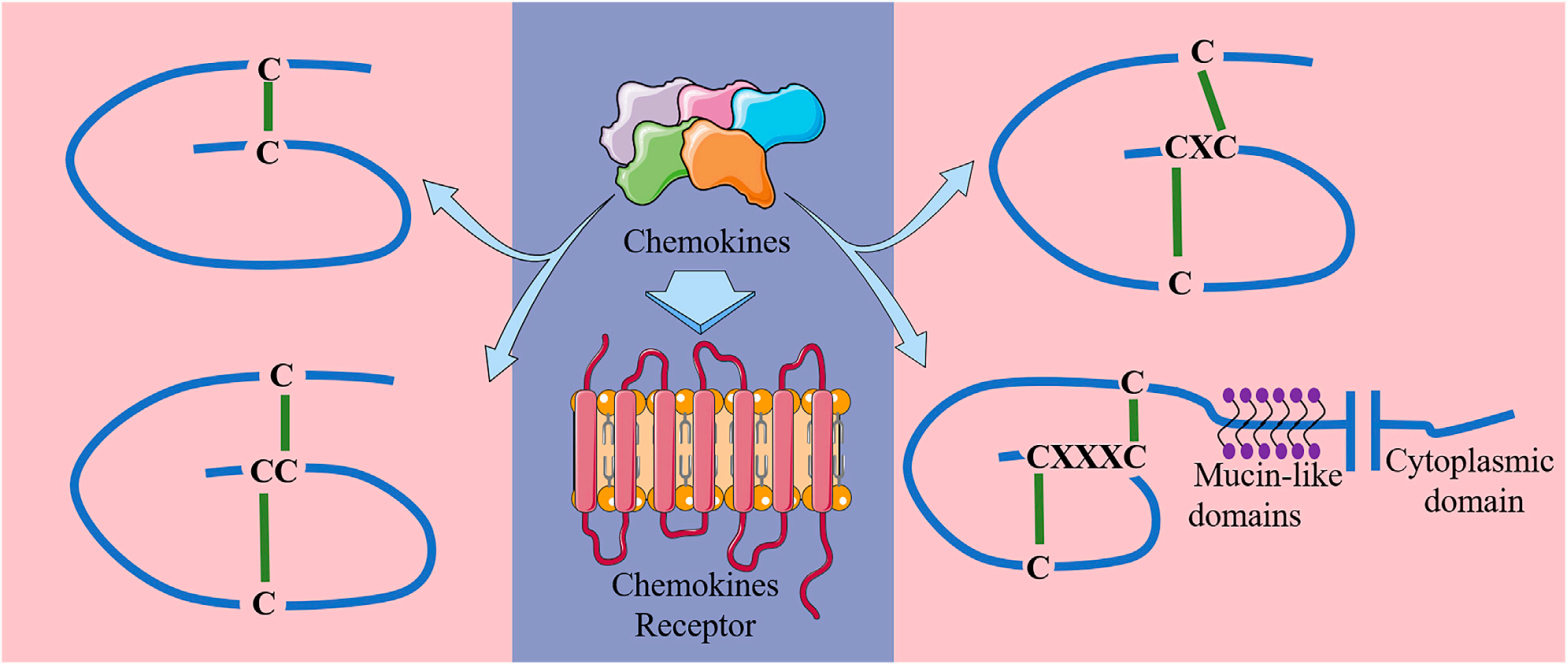
Main structures of the four classes of chemokines.

Chemokines can be classified into 4 classes in accordance to the number and location of their N-terminal pre-cysteines, namely class C, CC, CXC and CX3C (Miller and Mayo 2017) (Figure 1). Their biological effects are exerted through their specific interactions with chemokine receptors (Baggiolini 2001). Chemokine receptors are a kind of GPCRs that are selectively located in the membranes of target cells. To date, approximately 19 different chemokine receptors have been identified, which are also divided into four families based on the chemokine types they bind: CXCR, which binds to CXC chemokines; CCR, which binds to CC chemokines; CX3CR1, which binds to chemokine CX3CL1; and XCR1, which binds to chemokines XCL1 and XCL2 (Sharma 2010; Singh et al., 2011). Detailed chemical classifications are shown in Table 1.

**TABLE 1:** Classification of chemokines.

Classification	Chemokines	Chemokine Receptors	Reference
CC Chemokines	CCL1∼ CCL28	CCR	Singh et al. (2011)
CXC Chemokines	CXCL1∼ CXCL17	CXCR	Singh et al. (2011)
XC Chemokines	XCL1、XCL2	XCR1	Singh et al. (2011)
CX3C Chemokines	CX3CL1	CX3CR1	Sharma (2010), Singh et al. (2011)

All chemokines are small proteins that are composed of 70–100 amino acids with molecular weights of 8–10 kDa, and have four site-conserved cysteine residues to ensure their tertiary structure (Fernandez and Lolis 2002; Zlotnik and Yoshie 2012). The major function of chemokines is to induce directed migration of target cells, which can be attracted to specific tissues with increased chemokine concentration. In particular, chemokines can mediate leukocyte migration to respective locations during inflammation and homeostasis *in vivo* (Baggiolini 1998; Thelen and Stein 2008; Borroni et al., 2018). In this way, new light has been shed on antitumor therapy. For example, the CC-like chemokine CCL19 expressed in T cells of secondary lymphoid tissues and organs such as spleen and lymph nodes, is chemotactic to naive T cells and mature DC cells. Its specific receptor is CCR7, which is expressed not only on mature DC cells, macrophages and T cells (Förster et al., 2008; Comerford et al., 2013), but also in many tumor cells, such as colorectal (Xu et al., 2018), lung (Zhang et al., 2017), breast (Müller et al., 2001), and ovarian (Cheng et al., 2014). It has been shown that the interplay between the chemokine and its receptor could inhibit tumor proliferation, migration and invasion (Peng et al., 2015a; Xu et al., 2017; Zhou et al., 2020).

## Anti-Tumor Effect of Chemokines

### Involvement in Epigenetic Regulation

Epigenetics refers to heritable changes in the function of genes without alterations in their DNA sequences, ultimately leading to variation in the phenotype (Sapienza and Issa 2016). It is generally recognized that cancer epigenetics include modification of DNA and histones, regulation of non-coding RNA, chromatin remodeling, and nucleosome positioning (Dawson and Kouzarides 2012; Toh et al., 2017). Among the above-mentioned forms, methylation of DNA is the most well-studied epigenetic modification (Skvortsova et al., 2019). DNA methylation is a key epigenetic mechanism controlling gene expression, and in general, it inhibits gene transcription by shifting chromatin from a loose state, permissive for the active transcription, to a condensed state that prohibits the transcription (Moore et al., 2013; Zhang et al., 2014). The methylation pattern of DNA in the genome is catalyzed by DNA methyltransferases, which mainly involve DNMT1, DNMT3a, and DNMT3b. Among them, DNMT1 is the methyltransferase that plays a maintenance role, while DNMT3a and DNMT3b are mainly responsible for *de novo* methylation but also play a role in maintaining methylation (Bestor and Verdine 1994; Tajima et al., 2016).

It was shown that DNMT1-mediated DNA methylation and EZH2-mediated H3K27me3, in the enhancer region, suppressed CCL2 expression in SCLC cells, thereby enhancing tumor progression (Zheng et al., 2021). In addition, CXCL12 was down-regulated in gastric cancer tissues, accompanied by hypermethylation, and the reduced CXCL12 expression was closely associated with lymph node metastasis and histological grading, presumably playing a possible part in gastric cancer cell metastasis (Zhi et al., 2012). Similarly, upregulation of CXCR4 and downregulation of CXCL12 were observed in primary breast cancers. The hypermethylation in the CXCL12 promoter region in more than 50% of breast tumors was detected by methylation-specific PCR, and the expressions of DNMT1 and DNMT3b were distinctly higher in CXCL12-methylated breast cancers than in CXCL12-unmethylated breast cancers (Zhou et al., 2009). Additionally, studies by Ramos et al. and Dayer et al. corroborated the proposed perspective that the DNA methylation status of CXCR4 and CXCL12 genes could be used as biomarkers for breast cancer prognosis (Ramos et al., 2011; Dayer et al., 2018). The experiments of Peng et al. shown that EZH2-mediated H3K27me3 and DNMT1-mediated DNA methylation suppressed tumors and produced T helper 1 (TH1)-type chemokines CXCL9 and CXCL10, which could partially alter the T-cell landscape in cancer and may improve the clinical efficacy of cancer therapy (Peng et al., 2015b). In exploring the regulation of prostate cancer progression by hypermethylated in cancer 1 (HIC1) through epigenetic modifications, Zheng et al. were surprised to find that substantial methylation occurred within the HIC1 promoter and directly targeted the chemokine receptor CXCR7. Moreover, the CXCR7 promoter was negatively regulated by HIC1 (Zheng et al., 2013). In addition to CXCR7 studies, CXCL14 has also been well studied. Cao et al. reported that CXCL14 was frequently methylated in colorectal cancer, leading to downregulation of CXCL14 expression, and reversal of its expression inhibited the proliferation of colorectal cancer. Further experiments showed that CXCL14 inhibited the migration, infiltration and epithelial-to-mesenchymal transition (EMT) of colorectal cancer through suppressing the NF-κB signaling pathway (Cao et al., 2013). Furthermore, Tessema et al. identified that CXCL14 could be used as a typical target for epigenetic silencing in the development of lung cancer (Tessema et al., 2010). In addition, experiments of Song et al., using 5-aza-2-deoxycytidine as the demethylating agent to restore CXCL14 mRNA and protein expression, provided direct evidence for epigenetic regulation of chemokine expressions in tumor cells (Song et al., 2010).

### Involvement in Tumor Growth and Progression

Numerous experimental studies have established that chemokine signaling systems are involved in tumor growth and development via different mechanisms. For instance, interactions of chemokines with their receptors can directly activate signaling pathways, such as JAK/STAT and PI3K/AKT pathways, leading to cancer progression. More involved chemokines and their receptors acting on cancer are listed in Table 2.

**TABLE 2:** Effects on chemokines and their receptors in cancer.

Chemokine	Receptors	Tumor	Mechanism	Reference
CXCL12	CXCR4	Primary breast cancer	CXCR4 ↑, CXCL12 ↓ and the CXCL12 promoter region was hypermethylated	Zhou et al. (2009)
CXCL12	CXCR4	Breast cancer	DNA methylation	Ramos et al. (2011), Dayer et al. (2018)
CXCL13	CXCR5	Colorectal cancer	Activated a CXCL13/CXCR5/NFκB/p65/miR-934 positive feedback loop	Zhao et al. (2020)
CXCL13	CXCR5	Osteosarcoma	Regulated the phospholipase C beta, protein kinase C α, c-Src, and nuclear factor-κB signaling pathways	Liu et al. (2020)
CXCL11	CXCR3	Head and neck squamous cell carcinomas	Mediates tumor lymphatic cross-talk and inflammation-induced tumor	Kumaravel et al. (2020)
CXCL11	CXCR3	Liver tumor	Activated ERK1/2 through an autocrine signaling pathway	Zhang et al. (2019)
CXCL9	CXCR3	Tumor	Reinvigoration of CD8 T cell responses in response to PD-1 blocking tumor	Humblin and Kamphorst, (2019)
CCL2	CCR2	Hepatocellular carcinoma	Inhibits the recruitment of inflammatory monocytes, infiltration, and M2-polarisation of tumor-associated macrophages	Li et al. (2017)
CXCL1/8	CXCR2	Colorectal cancer	Recruited neutrophils to colorectal cancer tumor	Ogawa et al. (2019)
CXCL12	CXCR4	Epithelial ovarian cancer	Promote the proliferation, migration and invasion	Guo et al. (2013)
CXCL12	CXCR4	Gastrointestinal malignancies	Activation of G protein signaling kinases such as P13K/mTOR and MEK/ERK	Daniel et al. (2020)
CXCL12	CXCR7	Gastrointestinal malignancies	Activation of β-arrestin mediated signaling	Daniel et al. (2020)

The JAK/STAT pathway is the main signal transduction mechanism of various cytokines and growth factors, and has an important role in the regulation of biological processes such as cell growth, differentiation, proliferation, migration, and apoptosis. Studies have shown that multiple chemokines exert biological effects through JAK/STAT signaling pathway. It was found that CXCL1 was a direct target of miR-302e on cell proliferation, migration, invasion, and apoptosis in colorectal cancer, and the mechanism was correlated with CXCL1 expression regulated by miR-302e and the inactivation of the JAK-STAT signaling pathway (Chen et al., 2020). CXCR1, a receptor for CXCL1, is thought to be significantly related to poor prognosis in patients with NSCLC, and its high expression is mainly involved in signaling pathways such as JAK/STAT. Yang et al. used qRT-PCR and western blot experiments to show that overexpression of CXCR1 enhanced STAT5A expression, while knockdown of CXCR1 inhibited STAT5A expression (Yang et al., 2021). In addition, the combination of FKN and CX3CR1 could also activate the JAK/STAT signaling pathway and promote pancreatic cancer cell proliferation and migration (Huang et al., 2017). Meanwhile, chemokines can cause an imbalance between pro- and anti-apoptotic proteins in tumor cells. For example, chemokines down-regulated the expression of Bcl-2 and inhibited the activation of caspase-3 and caspase-9, thereby maintaining cancer cell survival and suppressing tumor cell apoptosis (Pang et al., 2015).

The PI3K/AKT pathway is an intracellular signaling pathway that responds to various extracellular signals and regulates a series of cellular function involved in metabolism, proliferation, cell survival, growth, angiogenesis, transcription, and protein synthesis. The above process is mediated through serine or threonine phosphorylation of a series of downstream substrates, and the key genes involved are PI3K and AKT (Jafari et al., 2019; Yang et al., 2019). Some chemokines binding to their receptors can activate the PI3K/AKT pathway, which in turn promotes a variety of biological functions and plays a critical part in many cancers. In melanoma and colorectal cancer, abnormally high CXCL5 expression activates PI3K/AKT signaling pathway and promotes PD-L1 expression, thereby creating an immunosuppressive microenvironment (Li et al., 2019). In addition, CXCL12 can regulate the expression of PTEN and affect colon cancer cell proliferation and invasion through PI3K/AKT signaling pathway (Ma et al., 2019). Interaction of CXCL13 with CXCR5 could also promote the growth and metastasis of colon cancer cells via PI3K/AKT pathway (Zhu et al., 2015). Furthermore, Chen et al. revealed that the expression of CCL26 in pancreatic cancer-associated fibroblasts was obviously increased by treating pancreatic adenocarcinoma with nab-paclitaxel. They further suggested that CCL26 enhanced the invasive ability of pancreatic adenocarcinoma cells through activation of PI3K/AKT/mTOR axis (Chen et al., 2021). Shen et al. found that CXCL8 induced the process of EMT through PI3K/AKT/NF-κB signaling pathway in colon cancer cells (Shen et al., 2017). Studies of Li et al. indicated that CCL25/CCR9 inhibited the apoptosis of NSCLS cell. The mechanism involved the activation of PI3K/AKT and the downstream upregulation of the anti-apoptotic proteins Bcl-2 and Bcl-xl and downregulation of the pro-apoptotic protein Bax (Li et al., 2015). Also, Ma et al. found that CXCL12 derived from fibroblasts significantly enhanced the secretion of CXCL6, and the synergistic effect of both chemokines could regulate colon cancer metastasis via PI3K/AKT/mTOR signaling pathway (Ma et al., 2017).

### Involvement in Tumor Invasion and Metastasis

A number of studies have confirmed the critical function of the chemokine axis in tumor metastasis. It has been reported that the expression of chemokine receptors on cancer cells can determine their sites of metastasis. These metastatic sites produce specific chemokines that advance the migration of moving cancer cells to “pre-metastatic ecological sites”, which provides favorable circumstances for the growth of metastatic cells (Murphy 2001; Adekoya and Richardson 2020). A variety of chemokines and chemokine receptors are correlated with cancer cell metastasis, and CXCL12/CXCR4 axis is a key representative system, which participates in the metastasis of various tumor cells (Teicher and Fricker 2010; Daniel et al., 2020). Besides, increased expression of CCR7 was remarkably associated with disease stage, grade, lymph node metastasis and neurovascular infiltration in breast cancer. Therefore, Vahedi et al. suggested that this biomarker could be used as a predictor of tumor metastasis and survival in patients (Vahedi et al., 2018). Acharyya et al. concluded that chemoresistance and metastasis are inextricably linked in cancer. CXCR2 blockers can break the CXCL1/2-S100A8/9 amplification circuit that causes chemoresistance, thus enhancing the chemotherapy effect in breast neoplasms, especially in metastasis (Acharyya et al., 2012). In addition, Cheng et al. devoted a large section to detail the role of chemokines and their receptors in the advancement and metastasis of lung cancer (Cheng et al., 2016). CCL18/PITPNM3 was proved to be associated with the migration, invasion and EMT processes in hepatocellular carcinoma by mediating the NF-κB signaling pathway (Lin et al., 2016). CCL28, a ligand for CCR3/CCR10, was also related to breast cancer growth and metastatic spread (Yang et al., 2017).

### Involvement in Angiogenesis

Chemokines and their respective receptors are considered to be key regulators of the tumor vascular system with a dual role in tumor angiogenesis. CXC chemokines are divided into two categories in accordance to the presence of ELR (Glu-Leu-Arg) motifs at the N-terminal end: ELR + chemokines and ELR–chemokines. ELR + CXC chemokines, such as CXCL1, CXCL2, CXCL3, CXCL5, CXCL6, CXCL7, and CXCL8, exert angiogenic effects by activating CXCR1 and CXCR2. In contrast, ELR–CXC chemokines, such as CXCL4, CXCL9, CXCL10, CXCL11, and CXCL14, are considered as angiogenesis inhibitors (Bosisio et al., 2014).

Tumor angiogenesis plays a prominent role in the process of tumor advancement. Chemokines can interact directly with specific chemokine receptors on vascular endothelial cells and act as regulators of tumor angiogenesis through endothelial cell signaling pathways, ultimately promoting migration and proliferation as well as endothelial cell survival (Keeley et al., 2011). The *in vivo* and *in vitro* experiments of Chen et al. showed that CXCL5 enhanced the angiogenic ability of colorectal cancer tumors in a CXCR2-dependent manner by a specific mechanism of activating the AKT/NF-κB/FOXD1/VEGF-A pathway. In addition, they found that CXCL5 also increased microvessel density in a subcutaneous xenograft tumor model in nude mice by overexpression treatment of CXCL5 (Chen et al., 2019). CCR6 has also been implicated in CCR6-mediated angiogenesis in colorectal cancer. Zhu et al. proposed that CCR6 promoted the secretion of vascular endothelial growth factor A (VEGF-A) through activation of the AKT/NF-κB pathway (Zhu et al., 2018). In studies of tumor angiogenesis in colorectal cancer, CXCL11 and CXCL12 have been shown to have a reciprocal regulatory role (Rupertus et al., 2014). In addition to acting directly on vascular endothelial cells, chemokines can also induce the proliferation of vascular endothelial cells through interacting with VEGF, which in turn promotes angiogenesis (Grunewald et al., 2006). Ping et al. found that CXCL12 could promote upregulation of VEGF expression through PI3K/AKT pathway in gliomas (Skvortsova et al., 2019). Moreover, their experimental results of using the CXCR4 antagonist AMD3100 or knocking out the CXCR4 gene showed that VEGF expression was reduced and tumorigenesis and angiogenesis was inhibited in a nude mouse lotus tumor model (Ping et al., 2011). In addition, CXCL8 and CXCL12-induced upregulation of VEGF expression resulted in the stimulation of angiogenic chemokine production (Kryczek et al., 2005; Martin et al., 2009).

On the other hand, chemokines also have the function of inhibiting tumor angiogenesis and endothelial cell proliferation. For instances, CCL19 could suppress tumor angiogenesis by promoting miR-206 expression dependently on CCR7, and thereby inhibiting the Met/ERK/Elk-1/HIF-1α/VEGF-A pathway. These results were also confirmed in a mouse angiogenesis model, where enhanced CCL19 expression inhibited angiogenesis in colorectal cancer *in vivo* (Xu et al., 2018). CXCL4L1 is a natural non-allelic variant of CXCL4. Struyf et al. proposed that CXCL4L1 was an effective anti-tumor chemokine, which can prevent the progression and metastasis of various tumors by inhibiting angiogenesis (Struyf et al., 2007). Furthermore, it has been postulated that CXCL4L1 exhibited vasopressor and chemotactic activity mediated by CXCR3 (Struyf et al., 2011). The CXCL12/CXCR4 biological axis is also closely related to tumor angiogenesis, and blocking this axis can inhibit tumor angiogenesis either by inhibiting VEGF or directly. Therefore, small molecule antagonists of CXCR4, such as ALX40-4C, AMD3100, and BKT140, have been used in tumor-related treatments (Sun et al., 2013). CXCL9, CXCL10, and CXCL11/CXCR3 are anti-tumor angiogenic factors, and the inhibition of tumor angiogenesis can be achieved via upregulating the expression of CXCL9, CXCL10, and CXCL11 (Billottet et al., 2013). In a retrospective analysis of 294 NSCLC patients taking Anlotinib, Lu et al. found a downregulation of serum CCL2 levels in patients. The results suggested that changes in serum CCL2 levels could be used as a marker to monitor clinical outcomes of patients with refractory advanced NSCLC (Lu et al., 2019). Other tumor angiogenesis-related chemokines and their receptors, such as CCL21/CCR7 and CXCL4, can be used as targets for anti-tumor angiogenesis therapy (Strieter et al., 1995; Somovilla-Crespo et al., 2013). Interestingly, the CC chemokines not only inhibit pathological angiogenesis but also maintain physiological angiogenesis (Ridiandries et al., 2017).

### Involvement in Tumor Microenvironment

Tumor microenvironment refers to not only the structure, function, and metabolism of tumor tissues, but also the internal environment of tumor cells themselves (Hinshaw and Shevde 2019; Vitale et al., 2019). The internal and external environment in which the tumor cells are located has a significant impact on the occurrence, growth, and metastasis of the tumor. Tumor microenvironment contains a diversity of cells and components, including lymphocytes, tumor-associated macrophages, cancer-associated fibroblasts, growth factors, cytokines, chemokines (Emon et al., 2018), which are of vital clinical significance for tumor prevention and treatment. Among them, chemokines and their receptors have attracted the attention of many researchers.

The interactions of chemokines and chemokine receptors can recruit immune cell subsets into the tumor microenvironment, and these interactions can regulate tumor progression and metastasis (Lee and Cho 2020). Marjorie et al. concluded that plasma CCL4 was positively correlated with inflammatory mediators and was associated with poor patient prognosis. They further suggested that high expression of CCL4 in colon cancer induces infiltration of tumor-associated macrophages (De la Fuente López et al., 2018). Zhang et al. observed that an increase in CCL3/6/8 led to the recruitment of myeloid cells, which restored immunosuppressive and pro-cancer effects. Further studies showed that depletion of regulatory T cells in pancreatic cancer led to differentiation of inflammatory fibroblast subpopulations, which in turn drove infiltration of bone marrow cells via CCR1, thus revealing a potential new therapeutic approach to alleviate immunosuppression in pancreatic cancer (Zhang et al., 2020). In addition, CCL2 plays a role in the recruitment of tumor-associated macrophages, which promote tumor phenotype generation as well as tumor cell invasion and angiogenesis (O'Connor and Heikenwalder 2021). The same is true for CCL24/27 (Lim 2021; Martínez-Rodríguez and Monteagudo 2021). CXCL13 and the receptor CXCR5 represent an emerging example of a chemokine signaling axis that demonstrates the ability to regulate tumor growth and progression. In addition, the CXCL13-CXCR5 axis may also indirectly regulate tumor growth by modulating non-cancerous cells in the tumor microenvironment, particularly immune cells (Hussain et al., 2021). The same applies to describe the critical role of the CXCL12-CXCR4 axis and the CCL2-CCR2 axis in the tumor microenvironment (Meng et al., 2018; Kadomoto et al., 2021). Han et al. proposed that blocking the CXCL8-CXCR1/2 axis alone or in combination with other immunotherapies would be a novel immunotherapeutic strategy (Han et al., 2021).

## Chemokines in Clinical Research and Applications

To date, the main means of treatment for tumors are still surgical treatment, radiation therapy, chemotherapy, and targeted therapy. Surgical treatment is mainly used for diagnosis and radical treatment, which can effectively relieve symptoms and improve survival. In recent years, with the improvement of radiotherapy equipment and the development of computer science, radiotherapy has been more and more widely used in clinical practice and has become an important means of treatment for comprehensive tumors. In addition, with the occurrence of new chemotherapeutic drugs, chemotherapy has acquired therapeutic importance. Although chemotherapy has greatly improved the survival rate of patients with advanced malignancies, it still needs to be combined with other treatments to improve the efficacy. Meanwhile, targeted therapy has gradually become vital for tumor treatment due to the development of genetic testing technology and small molecule targeted drugs. Targeted therapy enables selective, targeted, patient-friendly, and safer treatment to control tumor, thereby reducing the damage to normal tissues around the tumor. Therefore, it becomes more and more prominent in tumor treatment by virtue of its specificity and less toxicity.

Currently, chemokines and their receptors exhibit positive impacts in cancer biology, such as involvement in angiogenesis, metastasis, proliferation and invasion of cancer cells. Chemokines are also considered to be key influencers on disease progression and have a great effect on patients’ treatment and prognosis. In recent years, chemokines have been used as important therapeutic targets for cancer. Mogamulizumab (an anti-CCR4 antibody) and Plerixafor/AMD3100 (a CXCR4 antagonist) have been approved for the treatment of hematologic malignancies and being in clinical trials (Bule et al., 2021). In addition, Wsetermann et al. used CCL19-conjugated DNA vaccine for tumor control and showed that the combination of the two significantly inhibited tumor growth and prolonged the antitumor effect of the vaccine (Westermann et al., 2007). Subsequent studies have also confirmed that CCL19 can be used as an adjuvant for immunization with intradermal gene guns in a Her2/neu mouse tumor model, with enhanced vaccine efficacy (Nguyen-Hoai et al., 2012). Not coincidentally, the combination of CCL19/21 with CCL4 can also be used as an adjuvant for DNA vaccination in Her2/neu mouse tumor models (Nguyen-Hoai et al., 2016). In a recent clinical trial, Peng et al. found that the incorporation of CCL19 into chimeric antigen receptor (CAR)-engineered T cells dramatically improved the antitumor activity against human solid tumors, which has been in phase Ⅰ clinical trial (NCT03198546) (Pang et al., 2021). CCR2 in combination with FOLFIRINOX for advanced pancreatic ductal adenocarcinoma is in clinical phase II (NCT01413022) (Nywening et al., 2016). Additionally, CCR2 combined with Abraxane and Gemcitabine for metastatic pancreatic ductal adenocarcinoma is in phase Ib/II (NCT02732938) clinical trial (Noel et al., 2020). Other chemokines and receptors, such as CCL2 (Sandhu et al., 2013), CCR5 (Doi et al., 2019), and CXCR4 (Ghobrial et al., 2020), have also been used in combination with other drugs in a variety of cancers. More clinical trials involving chemokine therapy is listed in Table 3.

**TABLE 3:** Clinical trials involving chemokine therapy for cancer.

Molecule	Cancer	Status	Identifier	Reference
CCL2/Carlumab/CNTO 888	Ovarian and prostate cancer	Phase I	NCT00537368	Sandhu et al. (2013)
CCL2/Carlumab/CNTO 888	Metastatic prostate cancer	Phase II	NCT00992186	Pienta et al. (2013)
CCL2/Carlumab + docetaxel/gemcitabine/paclitaxel/carboplatin	Solid tumors	Phase Ib	NCT01204996	Brana et al. (2015)
CCL19 + chimeric antigen receptor (CAR)-engineered T cells + IL-7	Hepatocellular carcinoma	Phase I	NCT03198546	Pang et al. (2021)
CCR2 + FOLFIRINOX	Advanced pancreatic ductal adenocarcinoma	Phase II	NCT01413022	Nywening et al. (2016)
CCR2 + Abraxane + Gemcitabine	Metastatic pancreatic ductal adenocarcinoma	Phase Ib/II	NCT02732938	Noel et al. (2020)
CCR4/Mogamulizumab + nivolumab	Advanced/Metastatic solid tumors	Phase I	NCT02476123	Doi et al. (2019)
CCR4/Mogamulizumab	Peripheral T-cell lymphomas	phase II	NCT01192984	Ogura et al. (2014)
CCR4/Mogamulizumab + Durvalumab/Tremelimumab	Advanced solid tumors	Phase I	NCT02301130	Zamarin et al. (2020)
CCR5/Maraviroc	Refractory colorectal cancer	Phase I	NCT01736813	Halama et al. (2016)
CXCR4/Plerixafor	Refractory acute myeloid leukemia	Phase I/II	NCT00512252	Uy et al. (2012)
CXCR4/BL-8040 + Pembrolizumab	Pancreatic ductal adenocarcinoma	Phase IIa	NCT02826486	Bockorny et al. (2020)
CXCR4/Motixafortide + Pembrolizumab	Metastatic pancreatic cancer	phase II	NCT02826486	Bockorny et al. (2021)
CXCR4/Balixafortide + Eribulin	Metastatic breast cancer	Phase I	NCT01837095	Pernas et al. (2018)

## Conclusion and Prospect

**FIGURE 2:**
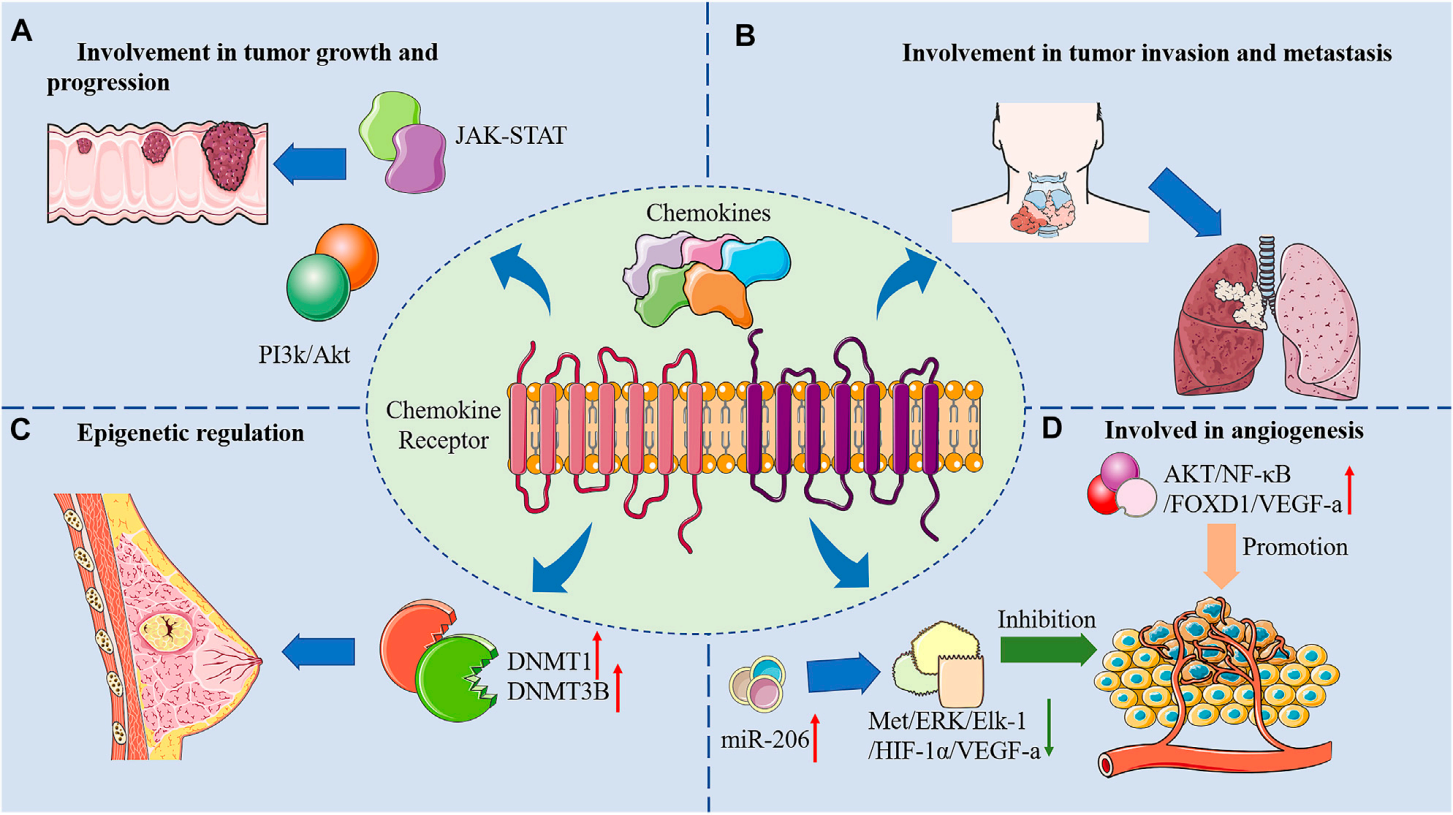
Mechanisms of anti-tumor effects of chemokines and its receptors.

Cancer is a life-threatening disease that imposes an economic burden on society. Due to its complexity and treatment resistance, diagnosing and curing cancer is a huge challenge. Despite recent advances in therapeutic strategies such as immunotherapy and targeted therapy, survival rates of cancer patients have not been reduced evidently. Chemokines are a large class of cytokines that coordinate the tropism of immune cell transport. They also participate in numerous cancer processes and serve as a critical part in the migration patterns of immune cells into tumors. The mechanisms of anti-tumor effects of chemokines and its receptors were briefly summarized in Figure 2.

The relationship between chemokines and/or chemokine receptors and tumors is complex and multifaceted, and has become a hot and difficult area in current tumor biology research. With the development of molecular biology, molecular immunology and related technologies, the role of chemokines/chemokine receptors system in tumor development and the related mechanisms have been gradually discovered. As a result, targeting these molecules may provide new strategies and means to targeted therapy of tumor. Through more in-depth research on tumor pathogenesis, more effective indicators for early diagnosis and determination of recurrence can be found, which will effectively reduce the morbidity and recurrence rate of cancer patients. A number of studies have suggested that chemokines can be involved in tumor development through a network of a variety of mechanisms. Also, chemokines are expected to become important indicators for tumor screening, diagnosis and monitoring in the future, because of the advantages of easy detection, low cost, and no surgery-related risks. Study of tumor-related chemokines has gradually turned into a research hotspot, although the specific biological properties and mechanism of action are still not fully elucidated. At present, some chemokines have been used as tumor diagnostic markers in clinical practice, and some chemokine-targeting drugs have entered various phases of clinical trial. Unfortunately, single-targeted chemokine therapeutic drugs have mostly ended up in failure. The new pathway of multicomplexer-based therapies will better help researchers discover new drugs with high effectiveness without negative impacts.

In summary, chemokines and their receptors are expected to become targets for new anti-tumor drugs and may provide a new approach to cancer therapy. As the mechanisms of interaction between chemokines and/or their receptors and cancer continue to be studied, chemokines and their receptors may also become predictors of cancer, which may then provide new strategies for targeted therapy and prevention of cancer.
